# The negative interplay between Aurora A/B and BRCA1/2 controls cancer cell growth and tumorigenesis via distinct regulation of cell cycle progression, cytokinesis, and tetraploidy

**DOI:** 10.1186/1476-4598-13-94

**Published:** 2014-04-28

**Authors:** Yan Wang, Ziliang Wang, Zihao Qi, Sheng Yin, Na Zhang, Yang Liu, Mingming Liu, Jiao Meng, Rongyu Zang, Zhen Zhang, Gong Yang

**Affiliations:** 1 Cancer Institute, Fudan University Shanghai Cancer Center, Shanghai 200032, China; 2 Department of Gynecological Oncology, Fudan University Shanghai Cancer Center, Shanghai 200032, China; 3 Department of Radiation Oncology, Fudan University Shanghai Cancer Center, Shanghai 200032, China; 4 Department of Oncology, Shanghai Medical College, Fudan University, Shanghai 200032, China; 5 Center Laboratory, The Fifth People’s Hospital of Shanghai, Fudan University, 128 Ruili Road, Shanghai 2000240, China

**Keywords:** Aurora A/B, BRCA1/2, Cell cycle, Cytokinesis, Tetraploidy, Tumorigenesis

## Abstract

It is well known that the activation of Aurora A/B (Aur A/B) or inactivation of BRCA1/2 induces tumor formation. Others and we have reported that the mutual suppression between Aur A/B and BRCA1/2 may manipulate cancer cell growth and tumorigenesis, however, the interactive regulation and mechanism between these molecules are still elusive. In this study, by consecutive silencing of Aur A/B or/and BRCA1/2 with specific shRNAs, we showed that, in BRCA2-deficient pancreatic cancer cell line Capan-1 and in ovarian cancer cell line OVCA433, Aur A/B and BRCA1/2 inversely regulated the expression of each other likely through proteasome-mediated proteolysis but not through gene transcription. Aur A/B and BRCA1/2 conversely regulated cell cycle progression mainly through control of p53 and cyclin A. Moreover, the disruption of Aur A/B blocked abnormal cytokinesis and decreased cell multinuclearity and chromosome tetraploidy, whereas the deprivation of BRCA1/2 promoted the abnormal cytokinesis and enhanced the cell multinuclearity and tetraploidy. Furthermore, we showed by animal assays that the depletion of Aur A/B inhibited tumor growth of both cell lines, while the knockdown of BRCA1/2 promoted the tumor growth. However, the concurrent silencing of Aur A/B and BRCA1/2 diminished the effects of these molecules on the regulation of cell cycle, cytokinesis, and tetraploidy, leading to the burdened tumor sizes similar to those induced by scrambled shRNA-treated control cells. In summary, our study revealed that the negative interplay between Aur A/B and BRCA1/2 inversely controls the cell proliferation, cell cycle progression, cell multinuclearity, and tetraploidization to modulate tumorigenesis.

## Introduction

In Aurora kinase family, Aurora A and Aurora B (simplified as Aur A and Aur B) are mostly studied members functioning as serine/threonine kinases regulating centrosome maturation, mitotic spindle assembly, and chromosome segregation [1]. It has been reported that the overexpression or amplification of Aur A/B drives chromosomal instability and induces tumor formation. Studies have shown that both Aur A and Aur B promote cell cycle progression [2,3]. Aur A is involved in cytokinesis, and overexpression of Aur A promotes abnormal cytokinesis and increases the number of polyploid cells [2,4]. Other studies suggest that the inactivation of Aur B promotes the completion of cytokinesis through abscission and prevents against tetraploidization [5].

Breast cancer susceptibility genes 1 and 2 (BRCA1/2) are known as tumor suppressor genes playing critical roles in DNA repair, cell cycle checkpoint control, and in maintenance of the genomic stability. Both BRCA1 and BRCA2 suppress the cell cycle progression [6-8]. Cancer cells in tissues of BRCA1 or BRCA2 mutation carriers are usually near tetraploid/polyploidy [9,10]. In mammary epithelial cells, blocking BRCA1 function leads to the aberrant mitosis with binuclear and tetraploidy [11]. Likewise, in murine embryo fibroblasts and cervical cancer cells, the absence of BRCA2 impairs the completion of cell division due to abnormal cytokinesis, leading to chromosomal polyploidization [12].

Increasing evidences have suggested that Aur A/B and BRCA1/2 may interplay to control the cell cycle, chromosome polyploidy, and tumorigenesis. For examples, Aur A physically binds to and phosphorylates BRCA1 at Ser308, leading to the abrogation of G2-M checkpoint [13], while BRCA1 can in return inhibit the activity of Aur A kinase through Gadd45a [14]. We have recently reported that Aur A and BRCA2 are mutually suppressed in ovarian cancer cells to control the RAS-associated genomic instability and tumorigenesis through the regulation of the cytokinesis and polyploidization [4]. Another study showed that the overexpression of BRCA2 in breast cancer cells suppresses the Aur A/B expression and reduces the number of polyploid cells [15]. Thus, Aur A/B and BRCA1/2 may interactively control the cell cycle progression, cytokinesis, polyploidization, and tumorigenesis. However, such an interactive regulation between Aur A/B and BRCA1/2 remains to be illustrated.

In this study, to explore novel interactive insights between Aur A/B and BRCA1/2, we used specific shRNAs to consecutively silence the expressions of Aur A/B and BRCA1/2 in pancreatic and ovarian cancer cell lines, and analyzed the cell proliferation, cell cycle progression, cytokinesis, chromosomal polyploidy, and tumorigenesis of resulting cells. Our results revealed that Aur A/B and BRCA1/2 are interactively suppressed to control the cancer cell growth and tumorigenesis through the regulation of the cell cycle progression, cytokinesis, and tetraploidy.

## Methods

### Cell lines and cell culture

Human pancreatic cancer cell line (Capan-1), ovarian epithelial cancer cell line (OVCA433), retroviral packaging cells (Phoenix amphotropic cells) and lentiviral packaging cells (293 T cells) were purchased from American Type Culture Collection (ATCC, America). Cells were maintained in Dulbecco’s modified Eagle’s medium (DMEM, Gibco), supplemented with 10% fetal bovine serum, 2 mM l-glutamine, penicillin (100 units/ml), and streptomycin (100 μg/ml). All cells were incubated at 37°C in an atmosphere of 5% CO_2_ and 95% air.

### Plasmids construction and cell transfection or viral infection

To silence the expressions of Aur A, Aur B, BRCA1 and BRCA2, the DNA oligonucleotides used to generate shRNAs against the open reading frames of their corresponding mRNAs were 5′-GUCUUGUGUCCUUCAAAUU-3′(Aur A shRNA), 5′- AGCCAUUUCAUCGUGGCGC-3′(Aur B shRNA), 5′-AAGUACGAGAUUUAGUCCG-3′ (BRCA1 shRNA) and 5′-ACAAUUACGAACCAAACCG-3′ (BRCA2 shRNA). pBabe/U6-neomycin-Aur Ai, pGIPz-puromycin-Aur Bi, pBabe/U6-zeocin-BRCA1i and pBabe/U6-blasticidin-BRCA2i were generated according to the previously reported method [4]. The control vectors were similarly constructed by directly inserting a scrambled shRNA (Scr) into the corresponding vectors [4].

Viruses from pBabe vectors were generated and harvested as described previously [4]. Viruses from pGIPz-puromycin-Aur Bi were generated using FuGENE 6 (Promega, San Luis Obispo, CA) and harvested according to manufacturer’s instruction. The resulting supernatant was used to infect target cells (Capan-1 and OVCA433) by using a method described before. Briefly, cells were infected twice for a total of 6 days (3 days for each infection) and the positive clones were selected with puromycin (200 ng/mL), neomycin (0.5-2.5 mg/ml), zeocin (100–750 μg/ml), or blasticidin (3–10 μg/ml) for 10–14 days to establish new stable cell lines.

The resulting cell lines were used later for various analyses, including immunoblotting, immunofluorescence and cell cycle.

### Real time fluorescence quantitative polymerase chain reaction

To analyze mRNA expression in cells, qRT-PCR analysis was performed as previously described [16]. Briefly, total RNA was isolated with Trizol reagent (Invitrogen, Carlsbad, CA). All RNAs were then reverse transcribed into cDNAs using the ExScript RT-PCR kit (TaKaRa, Japan) following the manufacturer’s instructions. Oligonucleotide primer pairs for Aur A, Aur B, BRCA1, BRCA2 and GAPDH are described in Additional file 1: Table S1. All amplifications and detections were carried out in the Applied Biosystems Prism 7900 system (Applied Biosystems, Foster City, CA) using the ExScript Sybr green QPCR kit (TaKaRa) and the following program: 1 cycle of 30 sec at 95°C followed by 40 cycles of (5 sec at 95°C, 20 sec at 60°C), followed by a 30-min melting curve collection, which was used to verify the primer dimers. Statistical analyses were performed using the 2^-△△CT^ relative quantification method. The assays were repeated three times in triplicate.

### Cell proliferation and colony formation assay

The cell proliferation was measured by 3-(4,5-Dimethylthiazol-2-yl)-2,5-Diphenyltetrazolium Bromide (MTT) Assay. 4 × 10^3^ cells for Capan-1 and 3 × 10^3^ cells for OVCA433 in 200 μl medium were incubated in 96-well culture plates. Cell growth was detected using MTT reagent with Synergy H4 Hybrid Reader. Briefly, the culture medium was removed at 1, 2, 3, 4, 5, 6, and 7 days, and 0.5 mg/ml MTT in 200 μl medium was added to each well and incubated for 4 h, followed by treatment with 150 μl of DMSO for 10 min. Data were collected by measuring the absorbance of optical density (OD) at 570 nm and subtracted from the background OD at 490 nm. The assays were repeated three times in triplicate.

For plate colony formation, 250 cells were seeded in 6-well plate. Duplicate cultures of each cell type were maintained at 37°C in a 5% CO_2_ atmosphere, and fresh medium was fed every 7 days. The number of colonies with >50 cells in each well was counted at 14 to 21 days. The assays were repeated three times.

### Cell cycle analysis

Cells (1–2 × 10^6^) were harvested, washed twice with 1 × PBS. The cells were fixed with 4 ml of cold 75% ethanol at −20°C overnight and then washed twice with 1 × PBS. The cells were then resuspended in 500 μl of 1× PBS and stained with 200 μl of propidium iodide (50 μl/ml; Sigma-Aldrich, Missouri, US) and 20 μl of RNase (1 mg/ml; Sigma-Aldrich, Missouri, US) in a 37°C water bath for 15 to 20 minutes. Cell cycles were determined by FAC Station (Beckman Coulter, FV500) and analyzed by using Kaluza® Flow Analysis Software and a published method. The assays were repeated three times.

### Immunoblotting

To analyze protein expression in cells, immunoblotting analysis was performed as previously described [2]. Antibodies against the following proteins were obtained from Santa Cruz Technology (California, US): Aur A (sc-25425), Aur B (sc-25426), BRCA1 (sc-6954), CDK2 (sc-163), CDK4 (sc-260), CDK6 (sc-177), cyclin D1 (sc-718), cyclin E (sc-247), cyclin A (SC-751). Antibodies against BRCA2 (19791-1-AP) and cyclin B1 (cs-4135) were obtained from Proteintech (Chicago, USA) and Cell Signaling Technology (Massachusetts, US), respectively. The detection of β-actin (A2228, Sigma Aldrich, St. Louis, MO) was used as a loading control. The secondary antibodies were F(ab)2 fragments of donkey anti-mouse immunoglobulin or of donkey anti-rabbit immunoglobulin linked to horseradish peroxidase from Cell Signaling Technology (Massachusetts, US). Immunoblotting reagents were from an electrochemiluminescence kit (Amersham Biosciences). To test whether the expression levels of proteins were regulated through proteasome-mediated degradation, cells were exposed with 20 μM MG132 (#S2619, Selleck Company, Texas, America) for 3 h and then harvested for Western blotting analysis.

### Immunofluorescence

Immunofluorescence staining was done according to a published protocol [2]. Primary antibodies against Aur A (sc-25425), Aur B (sc-25426), BRCA1 (sc-6954) and BRCA2 (sc-1818) were obtained from Santa Cruz Technology (California, US). DNA dye 4′,6-diamidino-2-phenylindole (DAPI) was obtained from Molecular Probes. The secondary antibodies used were the FITC-conjugated donkey anti-mouse IgG, cy3-conjugated donkey anti-rabbit IgG, Texas red-conjugated or FITC-conjugated donkey anti-goat IgG (Jackson ImmunoResearch Laboratory). All stained cells were examined and photographed with a Leica SP5 confocal fluorescence microscope.

### Analysis of metaphase chromosomes

Cells were cultured for 24 h and collected for chromosome preparation using standard procedures. Briefly, cells were exposed to colchicine (0.1 μg/mL for OVCA433 and 0.05 μg/ml for Capan-1) for 3 h, then subjected to hypotonic treatment (0.075 M KCl for 30 min at 37°C) and fixed in a mixture of methanol and acetic acid (3:1). After three changes of the fixative, the chromosome spreads were prepared by dropping the cell suspension onto cold slides, which were then air-dried. The slides were stained with Giemsa and examined for numerical abnormalities in the chromosomes. 3 slides and 30–36 metaphase spreads in each slide were analyzed for each cell line. The assays were repeated three times.

### Xenograft tumors in nude mice

To generate tumor growth in vivo, 5 × 10^6^ cells of each cell line were subcutaneously injected into 4- to 6-week-old BALB/c athymic nude mice (Department of Laboratory Animal, Fudan University). The animal experiments were approved by the Institutional Animal Care and Use Committee of Fudan University and performed following Institutional Guidelines and Protocols. Each cell line was bilaterally injected into six mice, for a total of 12 injections. The longest diameter “a” and the shortest diameter “b” of tumors were measured and the tumor volume was calculated with the use of the following formula: tumor volume (in mm^3^) = a × b^2^ × 0.52 [17], where 0.52 is a constant to calculate the volume of an ellipsoid. When a tumor reached 2.0 cm in diameter, the mouse were sacrificed and the tumors were weighed.

### Statistical analysis

Data were statistically analyzed with the Student *t* test. *P* < 0.05 was considered statistically significant.

## Results

### Interactive regulation of Aur A/B and BRCA1/2 in cancer cells

**Figure 1:**
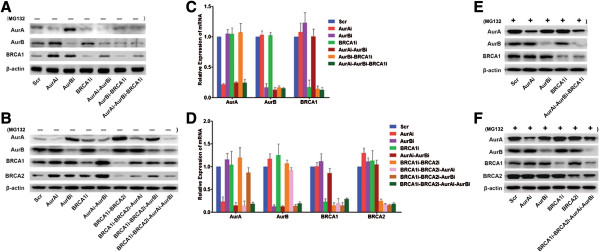
**Expression levels of Aur A/B and BRCA1/2. A-B**, Protein expressions detected by Western Blotting showing the interactive regulation of Aur A/B and BRCA1/2 before and after treatment with various shRNAs in BRCA2 deficient pancreatic cancer cells Capan-1 **(A)** and in ovarian cancer cells OVCA433 **(B)**. β-actin is used as a loading control. **C-D**, Relative mRNA levels of Aur A/B and BRCA1/2 in Capan-1 cells **(C)** and OVCA433 cells **(D)** detected by qRT-PCR compared with corresponding control cells (Scr cells). **E-F**, The protein levels of Aur A/B and BRCA1/2 in Capan-1 cells **(E)** and OVCA433 cells **(F)** treated with 20 μM MG132 for 3 h. β-actin is used as a loading control.

To examine the interaction between Aur A/B and BRCA1/2, we first analyzed the expression of these four proteins by Western blotting in cell lines treated with corresponding shRNAs. The results showed that the knockdown of Aur A (Aur Ai) increased the expression levels of Aur B and BRCA1 in Capan-1 cells, while the silencing of Aur B (Aur Bi) enhanced the expression levels of Aur A and BRCA1. The disruption of BRCA1 (BRCA1i) decreased the expression of Aur A, but not that of Aur B. Further knockdown of BRCA1 in cells with Aur B shRNA also downregulated the level of Aur A. BRCA1 was elevated in cells with concurrent silencing of Aur A and Aur B compared with scrambled shRNA-treated cells (Scr) (Figure 1A). These results suggested that Aur A/B and BRCA1 was interactively regulated in BRCA2 deficient Capan-1 cells. In ovarian cancer OVCA433 cells (Figure 1B), the interruption of Aur A did not alter the expression of Aur B, but the knockdown of Aur B enhanced the expression of Aur A. The silencing of Aur A or/and Aur B promoted the expressions of BRCA1 and BRCA2, compared with in scrambled shRNA-treated control cells. On the other hand, Aur A, but not Aur B, was increased in OVCA433-BRCA1i, OVCA433-BRCA1i-BRCA2i, and OVCA433-BRCA1i-BRCA2i-Aur Bi cells, compared with in scrambled shRNA-treated cells. Although BRCA1/2 was markedly reduced in OVCA433-BRCA1i-BRCA2i cells, a partially restored expression of BRCA1/2 was detected after Aur A and/or Aur B were depleted in such cells. Thus, a negative regulation loop between Aur A/B and BRCA1/2 was uncovered in both pancreatic and ovarian cancer cell lines.

To test whether Aur A/B and BRCA1/2 are regulated with each other through gene transcription, we first performed qRT-PCR to measure the mRNA levels of Aur A/B and BRCA1/2 in all cell lines treated with or without specific shRNAs. As shown in Figure 1C and D, the mRNA of each protein was reduced only in cell lines treated with its specific shRNA, but was not significantly altered in cell lines treated with non-isogenic shRNAs. Thus, these results suggested that the regulation of proteins between Aur A/B and BRCA1/2 might be at the post-translational level other than at the transcriptional level.

Because the proteasome-mediated protein degradation is one of the mostly characterized mechanisms in post-translational modification of many proteins, and increasing literatures have reported that the protein levels of Aur A/B and BRCA1/2 could be controlled via proteasome-mediated proteolysis [18-21], we treated cells with MG132, an inhibitor of the 26S proteasome, to test whether these proteins were essentially regulated through the classic protein degradation. As shown in Figure 1E and F, the inhibition of proteolysis by MG132 almost eliminated the divergences of protein expressions induced by non-isogenic shRNAs in cells, which is consistent with our previous result [4]. To sum up, the negative interplay of Aur A/B and BRCA1/2 proteins may be regulated mainly through proteasome-mediated protein degradation.

### Backward regulation of cell proliferation and colony formation by Aur A/B and BRCA1/2

**Figure 2:**
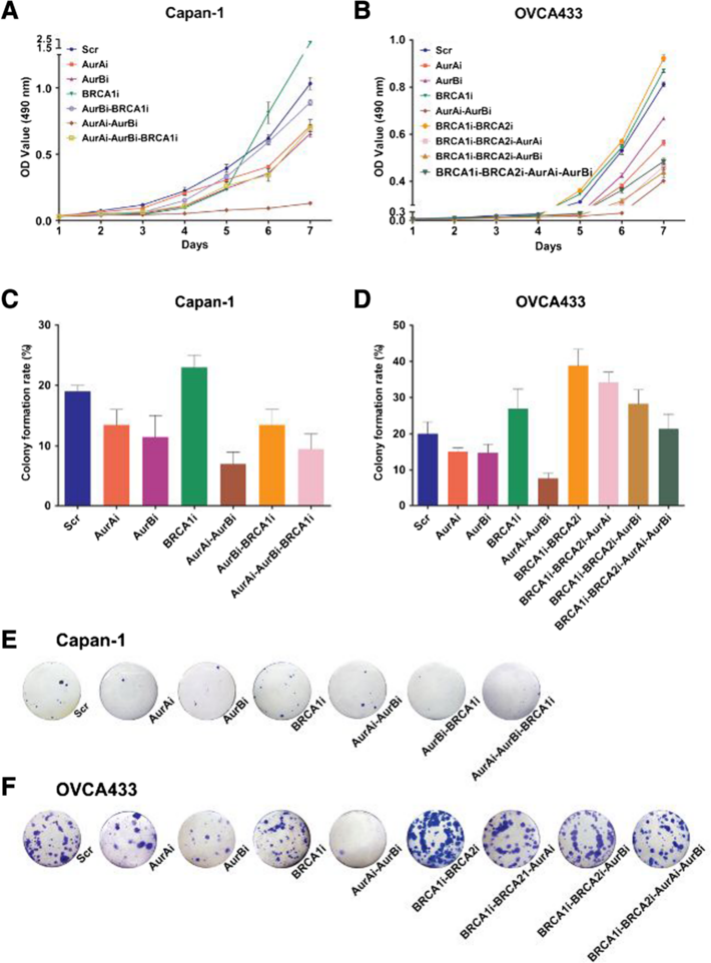
**Effects of Aur A/B and BRCA1/2 on cell proliferation and colony formation. A-B**, Alteration of cell growth curves before and after silencing of Aur A/B or/and BRCA1/2 in Capan-1 cells **(A)** and OVCA433 cells **(B)**. **C-D**, Colony formation rates of cells before and after disruption of Aur A/B or/and BRCA1/2. Data were collected from three independent experiments, and analyzed for statistic significance. Error bars = 95% CIs. **E-F**, Representative images of plate colony formation.

To investigate the effects of Aur A/B and BRCA1/2 on cell proliferation and colony formation, we first tested the growth curve of cells with or without silencing of Aur A/B or/and BRCA1. As shown in Figure 2A, compared with the corresponding control cells, silencing of Aur A or/and Aur B in Capan-1 cells restrained the cell growth, but silencing of BRCA1 promoted the cell growth. The concurrent depletion of Aur A and Aur B mostly inhibited the proliferation of Capan-1 cells. However, the knockdown of BRCA1 in Capan-1-Aur Bi or Capan-1-Aur Ai-Aur Bi cells converted the growth inhibition caused by the silencing of Aur B or Aur A/B. Similarly, in OVCA433 cells, the deprivation of Aur A or/and Aur B remarkably inhibited the cell growth (Figure 2B). Cells expressing BRCA1 shRNA proliferated more robustly than control cells, and the concurrent deprivation of BRCA1 and BRCA2 further augmented the cell proliferation. However, the silencing of Aur A or/and Aur B in OVCA433-BRCA1i-BRCA2i cells reduced the growth rate, compared with OVCA433-BRCA1i-BRCA2i and OVCA433-Scr cells. These data suggested that Aur A/B stimulates the cell growth, and BRCA1/2 prohibits the cell growth. The disrupting of Aur A and/or Aur B following the silencing of BRCA1 and/or BRCA2 can partially moderate the stimulated growth caused by the inhibition of BRCA1/2.

We then detected colony formation ability of various cell lines. Consistent with the proliferation assay, the colony number was reduced after silencing of Aur A and/or B, but was increased after the disruption of BRCA1/2 in both Capan-1 and OVCA433 cells in comparison with scrambled shRNA-treated control cells (Figure 2C-F). Introduction of Aur A and/or B shRNAs into cells along with BRCA1 or/and BRCA2 shRNAs in both pancreatic and ovarian cancer cells evidently reduced the number of colonies, compared with in cells expressing BRCA1 or/and BRCA2 shRNAs only. These results suggested that Aur A/B and BRCA1/2 may function as antagonists to inversely regulate the cell proliferation and colony formation, and that the effect of Aur A/B and BRCA1/2 on the cell proliferation and colony formation may be enhanced by the concurrent disruption of homologous partner proteins, but may be offset by the depletion of counterpart proteins.

**Figure 3:**
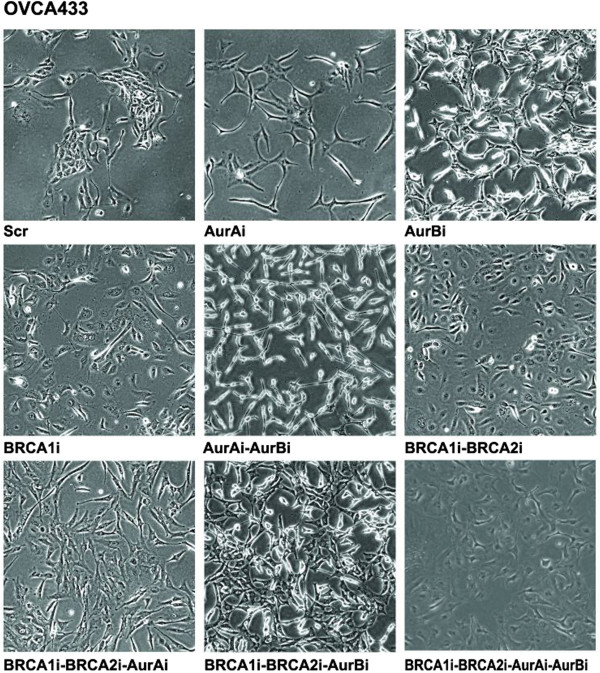
**Morphological changes of OVCA433 cells.** Selected images showing phenotypic changes of OVCA433 cells before or after the interruption of Aur A/B or/and BRCA1/2. Compared with OVCA433-Scr cells, the silencing of BRCA1 or/and BRCA2 induced cells scattered, whereas the interruption of Aur A and/or Aur B stimulated cells thinner and more dispersed compared with control cells. The disruption of Aur A and/or Aur B following double knockdown of BRCA1 and BRCA2 stimulated cells growing thinner and flatter than cells with intact Aur A and/or Aur B expressions. However, cells with the concomitant silencing of four proteins turned oblate. The images were taken at 200 × magnification by an Olympus microscope.

Interestingly, we found that the knockdown of Aur A/B or/and BRCA1/2 diversified the cellular morphology of OVCA433 cells. As shown in Figure 3, cells with the silenced expression of Aur A or/and Aur B appeared thinner and more dispersed than scrambled shRNA-treated (OVCA433-Scr) control cells viewing plump and congregate. Cells expressing BRCA1 shRNA or both BRCA1 and BRCA2 shRNAs still appeared plump, but scattered. OVCA433-BRCA1i-BRCA2i cells with Aur A or Aur B shRNAs appeared thinner and flatter than those without Aur A or/and Aur B shRNAs. However, cells with the synchronized silencing of the four proteins turned oblate. The above results indicate that Aur A/B and BRCA1/2 may potentially regulate cytoskeleton-associated proteins, leading to phenotypic alterations of cancer cells. However, the morphological changes of Capan-1 cells were not observed before or after the silencing of Aur A/B and/or BRCA1 (data not shown).

### Converse control of cell cycle progression by Aur A/B and BRCA1/2

**Figure 4:**
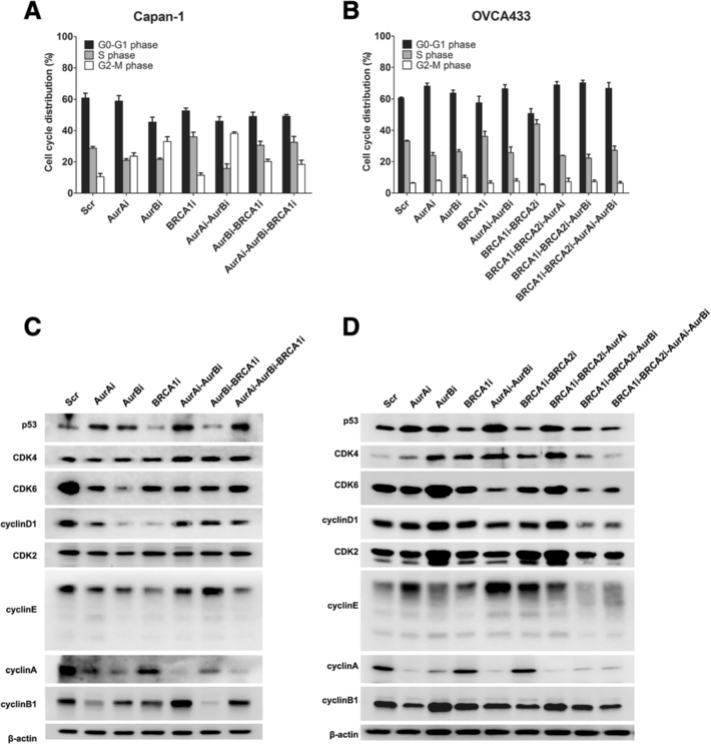
**Regulation of cell cycle progression and cell cycle-associated proteins by Aur A/B and BRCA1/2. A**-**B**, Cell cycle distributions detected by flow cytometry before or after silencing of Aur A/B and/or BRCA1/2. **C**-**D**, Alteration of cell cycle regulatory proteins tested by immunoblotting. Inverse regulation of p53 and cyclin A by Aur A/B and BRCA1/2 in both Capan-1 and OVCA433 cells is conceived. β-actin is used as a loading control.

Since Aur A/B and BRCA1/2 participate in cell cycle control, we examined the effects of interplay between Aur A/B and BRCA1/2 on cell cycle progression. Compared with in Capan-1-Scr cells, the silencing of Aur A and/or B decreased the number of cells in S phase, but increased the number of cells in G2-M. The disruption of BRCA1 reduced the cell population in G0-G1 phase and raised the number of cells in S phase, but did not alter the cell population in G2-M phase. However, the silencing of BRCA1 in Capan-1-Aur Bi or Capan-1-Aur Ai-Aur Bi cells perceptibly stimulated the number of cells in S phase, but reduced the number of cells in G2-M phase (Figure 4A). In OVCA433 cells, the disruption of Aur A or/and B augmented the number of cells in G0-G1 phase, and diminished the number of cells in S phase, but did not affect the cell population in G2-M phase. The combined silencing of Aur A and Aur B did not yield synergistic effects on the cell cycle progression, compared with the solo silencing of Aur A or Aur B. Compared with control cells, the silencing of BRCA1 promoted the cell population in S phase, and limited the percentage of cells in G2-M phase, but did not induce any changes of cell proportion in G0-G1 phase. The concurrent silencing of BRCA1 and BRCA2 profoundly reduced the number of cells in G0-G1 phase, and enhanced the cell population in S phase. However, the depletion of Aur A and/or Aur B in BRCA1 and BRCA2 shRNAs-treated cells increased the number of cells in G0-G1 phase and lessened the number of cells in S phase, compared within cells without Aur A or/and Aur B shRNAs (Figure 4B).

To validate the cell cycle alteration induced by the interruption of Aur A/B and BRCA1/2, we tested the major proteins involved in cell cycle regulation by Western blotting. Compared with in Capan-1-Scr (control) cells, the silencing of Aur A or Aur B enhanced the expression of p53, and attenuated the expressions of CDK6, cyclins D1, E, A, and B1 (Figure 4C). The concurrent silencing of Aur A and Aur B up-regulated the p53 expression, down-regulated the levels of CDK6, cyclins D1, E, and A, but enhanced the CDK4 and cyclin B1 expressions. The interruption of BRCA1 in Capan-1 cells decreased the expressions of p53, CDK6, cyclins D1, E, and B1 compared with in control cells. The disruption of both BRCA1 and Aur B blocked the expressions of p53 and cyclin B1, while lifted the expressions of CDK4, CDK6, CDK2, cyclins D1, E, and A in the resulting cells, compared with in cells expressing Aur B shRNA alone. However, the depletion of BRCA1 in Capan-1-Aur Ai-Aur Bi cells elevated the expressions of CDK6, but dropped the expressions of p53, cyclins D1, E, and B1 in Capan-1-Aur Ai-Aur Bi-BRCA1i cells. Collectively, the knockdown of Aur A/B mainly promoted the p53 expression but lessened the levels of most CDKs and cyclins to restrain the cell cycle progression of Capan-1 cells, while the disruption of BRCA1 inhibited p53 to promote the cell cycle progression.

The effects by the knockdown of Aur A/B or/and BRCA1/2 on the expressions of above proteins in OVCA433 cells (Figure 4D) were similar to those in Capan-1 cells. Compared with in OVCA433-Scr cells, the silencing of Aur A lifted the levels of p53, CDK4, cyclin E, but reduced the levels of cyclins A and B1 in OVCA433-Aur Ai cells. The silencing of Aur B promoted the expressions of p53, CDK4, CDK6, CDK2, and cyclin B1, but inhibited the expression of cyclin A in OVCA433-Aur Bi cells. Further silencing of Aur B in OVCA433-Aur Ai cells strengthened the levels of p53, CDK4, and cyclin E and B1, but largely reduced the levels of CDK6 in OVCA433-Aur Ai-Aur Bi cells. On the other hand, compared with in OVCA433-Scr cells, the depletion of BRCA1 did not alter the p53 expression, but up-regulated the CDK4 expression in OVCA433-BRCA1i cells. Further deleting of BRCA2 in OVCA433-BRCA1i cells increased the cyclin E expression, but slightly decreased the cyclin B1 expression the levels of p53, CDK4, CDK6, cyclin D1, and cyclin A were not conceivably changed. However, the disruption of Aur A in OVCA433-BRCA1i-BRCA2i cells enhanced the levels of p53, CDK4, CDK6, cyclin D1, CDK2, and cyclin B1, but blocked the expressions of cyclins E and A. The knockdown of Aur B in OVCA433-BRCA1i-BRCA2i cells diminished the levels of CDK4, CDK6, CDK2, cyclins D1, E, and A, but did not alter the level of p53. Moreover, the concurrent depletion of Aur A and B in OVCA433-BRCA1i-BRCA2i cells reduced the levels of CDK4, CDK6, CDK2, cyclin D1, E and A, which converted the changes of these proteins, suggesting that these proteins may be regulated by the interplay between Aur A/B and BRCA1/2.

To sum up, the expression of the cell cycle regulatory proteins including p53, CDK4, CDK6, cyclin D1, CDK2, cyclin E, cyclin B1, and cyclin A could be effectively regulated by Aur A/B and BRCA1/2 to control the cell cycle progression, and Aur A/B and BRCA1/2 may modulate the cell cycle fundamentally through p53 and cyclin A.

### Counter impacts of Aur A/B and BRCA1/2 on cell multinuclearity and tetraploidy

**Figure 5:**
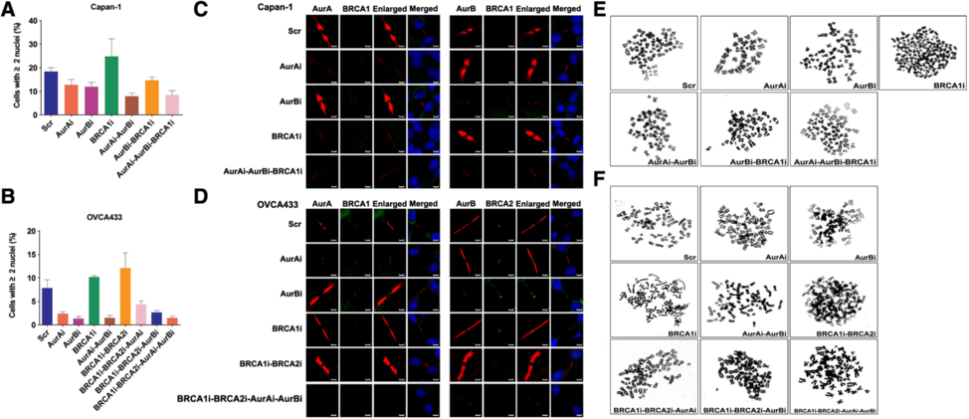
**Regulation of polyploidy and cytokinesis by Aur A/B and BRCA1/2. A**-**B**, Quantification of cells with multiple nuclei before or after the depletion of Aur A/B and BRCA1/2. Error bars = 95% CIs, indicating data were collected from three independently repeated experiments. **C**-**D**, Representative images showing the localization and accumulation of Aur A/B and BRCA1/2 at the midbody during cytokinesis (×1000). Blue dye DAPI indicates nucleus. Scale bars, 5 μm for the merged images and 1 μm for other images. **E**-**F**, Representative images of karyotypes before and after the silencing of Aur A/B or/and BRCA1/2 in Capan-1 cells **(E)** and OVCA433 cells **(F)**.

Counts of cells with two or more nuclei showed that the number of cells with multinuclearity was lower in Capan-1-Aur Ai/Aur Bi or OVCA433-Aur Ai/Aur Bi cells than in Capan-1-Scr or OVCA433-Scr cells. Cells with the concomitant silencing of Aur A and Aur B in Capan-1- or OVCA433-Aur Ai-Aur Bi cells appeared with more multinuclearity than those with the solo depletion of Aur A or Aur B in Capan-1- or OVCA433-Aur Ai/Bi cells (Figure 5A-B). The deprivation of BRCA1 increased the proportion of cells with multiple nuclei in Capan-1- or OVCA433-BRCA1i cells. However, compared with in Capan-1-Aur Ai-Aur Bi cells, the cell multinuclearity was not markedly increased in Capan-1-Aur Ai-Aur Bi-BRCA1i cells (Figure 5A). The diminution of BRCA2 in OVCA433-BRCA1i-BRCA2i cells enhanced the proportion of cells with multiple nuclei compared with in OVCA433-BRCA1i cells. However, the intervention of Aur A or/and Aur B in OVCA433-BRCA1i-BRCA2i cells salvaged the functional effects of BRCA1/2 on suppression of the cell multinuclearity in OVCA433-BRCA1i-BRCA2i-Aur Ai, OVCA433-BRCA1i-BRCA2i-Aur Bi, OVCA433-BRCA1i-BRCA2i-Aur Ai-Aur Bi cells (Figure 5B).

Abscission is the last step of cytokinesis and the restrained abscission causes cytokinesis failure, resulting in cells with the multinuclearity and tetraploidy. Since Aur A/B and BRCA1/2 have been reported to be accumulated at midbody during the cytokinesis of late mitosis [4,5,22,23], we detected these proteins by immunofluorescence. As shown in Figure 5C, both Aur A and Aur B were localized at the midbody during the late telophase in Capan-1 cells, but only a few of BRCA1 protein was observed at the midbody. The diminution of Aur A or BRCA1 slightly increased the expression of Aur B at the midbody. Aur A/B and BRCA1/2 co-localized at the midbody during the late telophase, but the staining of BRCA1 was faint in OVCA433 cells. The interruption of Aur A/B enhanced the BRCA2 accumulation at the midbody, but did not conceivably modify the staining of BRCA1. On the other hand, the revoking of BRCA2 following the BRCA1 knockdown dramatically enhanced the Aur A/B accumulation at the midbody (Figure 5D). Thus, Aur A/B and BRCA2 may inversely regulate cytokinesis to control the cell tetraploidization.

Analysis of chromosome karyotyping showed that the silencing of Aur A and/or B reduced the proportion of tetraploid cells in both Capan-1 and OVCA433 cells than that in control cells. The disruption of BRCA1 or/and BRCA2 in Capan-1 or OVCA433 cells resulted in more tetraploid than did in control cells. The depletion of BRCA1 in Capan-1-Aur Ai-Aur Bi cells promoted tetraploidy in Capan-1-Aur Ai-Aur Bi-BRCA1i cells. Additional deprivation of Aur A and/or Aur B in OVCA433-BRCA1i-BRCA2i cells reduced the level of tetraploidy in OVCA433-BRCA1i-BRCA2i-Aur Ai, OVCA433-BRCA1i-BRCA2i-Aur Bi and OVCA433-BRCA1i-BRCA2i-Aur Ai-Aur Bi cells (Table 1, Figure 5E-F).

**Table 1:** Aur A/B and BRCA1/2 mediate cell polyploidy

	**Cell lines**^**1**^	**Diploidy cells (%)**	**Tetraploidy cells (%)**	**N**^**2**^
Capan-1	*Scr*	78.6 ± 3.2	16.2 ± 3.3	302
	*Aur Ai*	82.3 ± 3.9 (*S↑)	11.6 ± 5.1 (*S↓)	300
	*Aur Bi*	87.3 ± 5.9 (*S↑)	10.6 ± 4.6 (*S↓)	302
	*BRCA1i*	72.8 ± 6.1 (*S↓)	22.3 ± 6.4 (*S↑)	303
	*Aur Ai-Aur Bi*	89.0 ± 4.7 (*S↑)	6.4 ± 3.9 (*S↓)	306
	*Aur Bi-BRCA1i*	83.4 ± 5.0 (*S↑)	9.3 ± 6.7 (*S↓)	303
	*Aur Ai-Aur Bi-BRCA1i*	78.0 ± 4.8 (*AB↓)	10.0 ± 4.7 (*AB↑)	299
OVCA433	*Scr*	88.9 ± 4.5	11.1 ± 4.5	287
	*Aur Ai*	95.0 ± 5.0 (*S↑)	5.0 ± 5.0 (*S↓)	298
	*Aur Bi*	95.0 ± 2.1 (*S↑)	5.0 ± 2.1 (*S↓)	302
	*BRCA1i*	80.0 ± 6.3 (*S↓)	20.0 ± 6.3 (*S↑)	295
	*Aur Ai-Aur Bi*	97.8 ± 2.4 (*S↑)	2.2 ± 2.4 (*S↓)	300
	*BRCA1i-BRCA2i*	77.8 ± 6.1 (*S↓)	22.2 ± 6.1 (*S↑)	290
	*BRCA1i-BRCA2i-Aur Ai*	90.0 ± 4.2 (*B1B2↑)	10.0 ± 4.2 (*B1B2↓)	294
	*BRCA1i-BRCA2i-Aur Bi*	84.6 ± 4.7 (*B1B2↑)	14.4 ± 4.9 (*B1B2↓)	290
	*BRCA1i-BRCA2i-Aur Ai-Aur Bi*	91.2 ± 4.8 (*B1B2↑)	7.8 ± 5.6 (*B1B2↓)	290

^1^For each cell line, 30–36 cells in metaphase in every slide were examined. The assays were repeated three times. The increase or decrease of cells with diploidy or tetraploidy was indicated as “↑” or “↓”, respectively. ^*^p < 0.05. S, AB or B1B2 represent cells expressing Scr, Aur Ai-Aur Bi or BRCA1i-BRCA2i used for statistical analysis.

^2^ N represents the total number (N) of cells karyotyped for each cell line.

### Opposite function of Aur A/B and BRCA1-2 in tumorigenesis

**Figure 6:**
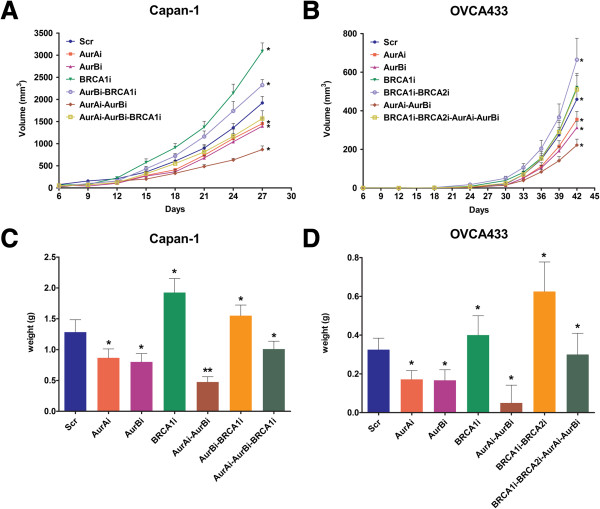
**Xenograft tumor growth in animals. A**-**B**, The mean volume of tumors burdened in mice receiving Capan-1 or OVCA433 cells expressing scrambled shRNA or different shRNAs against Aur A/B or BRCA1/2. Error bars = 95% CIs. *P < 0.05. **C**-**D**, The average weight of each tumor formed in different groups of mice. Error bars = 95% CIs. *P < 0.05, **P < 0.01.

To test how Aur A/B and BRCA1/2 affect tumor growth, we injected Capan-1 and OVCA433 cells expressing various shRNAs along with their control cells into nude mice and analyzed the tumor growth. As shown in Figure 6A and C, the knockdown of Aur A or Aur B in Capan-1 cells inhibited the tumor growth, but the silencing of BRCA1 alone promoted the tumor growth in mice, compared with relative control cells. Tumors burdened in animals injected with Capan-1-Aur Ai-Aur Bi cells were smaller than any other tumors derived from Capan-1 cells treated with shRNAs. The volumes of tumors induced with Capan-1-Aur Bi-BRCA1i or Capan-1-Aur Ai-Aur Bi-BRCA1i cells were bigger than those with Capan-1-Aur Bi, or Capan-1-Aur Ai-Aur Bi cells. Similarly, the tumors generated with OVCA433 cells treated with Aur A and/or Aur B shRNAs developed more slowly, but those generated with cells expressing BRCA1 and/or BRCA2 shRNAs advanced much faster than tumors produced with control cells (Figure 6B and D). Among tumors derived from OVCA433 cells, those inoculated with OVCA433-BRCA1i-BRCA2i cells were the most robust, but those derived from OVCA433-Aur Ai-Aur Bi cells were the slowest. These results suggest that Aur A/B and BRCA1/2 manage tumorigenesis in an opposite manner, and that the effects on tumorigenesis by disrupting one protein can be synergized by depleting the other homolog or can be diminished by silencing one of the counterpart proteins.

## Discussion

The control of cell cycle in normal cells plays a key role in maintaining genetic fidelity during cell division. Any errors occurred during cell cycle progression may cause chromosome abnormalities, leading to cell aneuploidy or polyploidy, and subsequent tumorigenesis. It has been reported that the elevated Aur A promotes G1-S and G2-M transition [2], while silencing of Aur B results in acute cell cycle arrest in G1 phase [24]. Phosphorylation of BRCA1 at S308 by Aur A in the M phase is an early event necessary for G2-M transition [13]. Loss of BRCA1/2 leads to override of M phase, multinucleation and tetraploidy/polyploidy [9,10]. We showed that, in this study, the silencing of Aur A/B suppressed overall cell cycle progression mainly through G1-S and G2-M transition, while the disruption of BRCA1/2 mainly promoted cell cycle progression through accelerated G1-S and G2-M transition, suggesting that Aur A/B and BRCA1/2 negatively regulate G1-S and G2-M transitions to control cell cycle progression. Furthermore, we found that the expression of p53 was negatively regulated by Aur A/B, but positively regulated by BRCA1/2, which was consistent with previous studies [25,26], indicating that p53 might be the focused target of both Aur A/B and BRCA1/2 through which to modulate cell cycle progression and tetraploidization. Moreover, studies have shown that cyclin A is essential for the G1-S and G2-M transitions [27], and that cyclin A availability is the rate-limiting step for entry into mitosis [28]. We found that the disruption of Aur A/B down-regulated cyclin A expression, but the silencing of BRCA1/2 up-regulated cyclin A. Therefore, cyclin A may be another mediator regulated conversely by Aur A/B and BRCA1/2 to control cell proliferation, cell cycle progression, and tumorigenesis.

Complete mitosis is composed of nuclei division and cytoplasm separation - cytokinesis. The last step of cytokinesis is the abscission of midbody, the failure of which is associated with delayed cytokinesis and ploidy changes [29]. Inactivation of Aur B promotes completion of cytokinesis by abscission to suppress tetraploidization [5]. BRCA2 interacts with many abscission factors at the midbody, and the disruption of these abscission factors results in increased cytokinetic defects [22]. We previously reported that Aur A inversely regulates BRCA2 at the midbody during cytokinesis to promote polyploidy [4]. In this study, we showed that Aur A/B and BRCA2 were co-localized at midbody and conversely regulated the counterparts during late mitosis, indicating that the interplay of Aur A/B and BRCA2 may manipulate cytokinesis to keep a proper segregation of two daughter cells from polyploidy. Additionally, as reported, although no BRCA1 staining was observed at the midbody in immunofluorescence slides of cervical cancer cells HeLa [30], evident localization of BRCA1 was found in the midbody area during cytokinesis in immunoelectron-microscopic sections of breast cancer cells SKBR3 [23]. In this study, the staining of BRCA1 at the midbody of mitotic Capan-1 cells was not strong, and the knockdown of Aur A/B did not fortify the accumulation of BRCA1 at the midbody, indicating that the role of BRCA1 at the midbody may be less valuable. However, we did find that the silencing of BRCA1 promoted cell tetraploidization, indicating that BRCA1 may regulate tetraploid through a different way, for example the regulation of centrosome. More importantly, the concurrent silencing of Aur A, Aur B, BRCA1, and BRCA2 did not induce complete cytokinesis failure in both Capan-1 and OVCA433 cells, implying that some other factors such as survivin and HIPK2 It is well-known that the proteasome-mediated degradation may still function to maintain cytokinesis [31,32].

Is one of the possible mechanisms in the post-translation regulation proteins. The mounting studies have evidenced that the expression levels of Aur A/B and BRCA1/2 may be also controlled through ubiquitin-mediated protein degradation. For instances, FBXW7, a component of E3 ubiquitin ligase, can target both Aur A and Aur B for ubiquitination and subsequent degradation [18,19]. BRCA1 forms a heterodimer with BRCA1-associated RING domain 1 (BARD1) and exhibits the E3 ubiquitin ligase activity to target other proteins [33], while the Aur A kinase functions to inhibit the E3 ubiquitin ligase activity of BRCA1 [34]. Moreover, BRCA1/2 may interact with Aur B through the BRCA1-associated ring domain protein 1 (BARD1) to modulate the mitotic spindle formation, cytokinesis, aneuploidy and genomic instability [30]. BARD1β, an isoform of BARD1 deficient of RING domain, stabilizes Aur B and forms a complex with BRCA2 and Aur B [30]. In this study, by qRT-PCR and cell treatment with MG132, we showed that the interactive regulation of protein expressions between Aur A/B and BRCA1/2 was mainly controlled through the proteasome-mediated proteolysis although the detailed mechanism may need more investigations.

In conclusion, the interplay of Aur A/B and BRCA1/2 constitutes a regulatory network in cancer development. A schematic diagram in Additional file 2: Figure S1 is to illustrate that Aur A/B and BRCA1/2 were negatively regulated between each other likely through proteasome-mediated proteolysis (PMP) to control cell proliferation, cell cycle progression, cytokinesis, tetraploid, and eventually tumorigenesis of cancer cells. Molecules including p53 and cyclin A may be the major mediators inversely regulated by imbalanced expressions of Aur A/B and BRCA1/2 in cancer cells. Although the interactive regulation between Aur A/B and BRCA1/2 may lie in different pathways such as proteolysis as we reported [4], this study may have provided some novel insights that the negative interplay between Aur A/B and BRCA1/2 plays an essential role during cancer development. The results of this study may be used to improve the efficacy of the current therapeutic methods in cancers specifically with amplified Aur A/B and inactivated BRCA1/2.

## Competing interests

The authors declare that they have no competing interests.

## Authors’ contributions

YW, ZW, RZ, ZZ and GY conceived the project, and designed the experiments. YW, ZQ, SY, NZ, YL, ML and JM performed the experiments and analyzed the data. All authors contributed to data interpretation. YW and GY wrote the manuscript with input from all other authors.

## Supplementary Material

Additional file 1: Table S1 Sequences of oligonucleotide primer pairs for qPCR. Click here for file

Additional file 2: Figure S1 A schematic diagram illustrates how Aur A/B and BRCA1/2 regulate cell cycle progression and cytokinesis to modulate tetraploidy and tumorigenesis. PMP: proteasome-mediated proteolysis. Click here for file
