# Supplementary material for: The negative interplay between Aurora A/B and BRCA1/2 controls cancer cell growth and tumorigenesis via distinct regulation of cell cycle progression, cytokinesis, and tetraploidy
Source: Mol Cancer. 2014 Apr 28;13:94. doi: 10.1186/1476-4598-13-94 (PMC4028103; doi:10.1186/1476-4598-13-94)
Supplement: Additional file 1: Table S1 — Sequences of oligonucleotide primer pairs for qPCR. [file 1476-4598-13-94-S1.doc]

**Supplementary Table1. Sequences of oligonucleotide primer pairs for qPCR**

| Gene | Primer sequence | References |
| --- | --- | --- |
| AurA | Forward: 5’- GGAGAGCTTAAAATTGCAGATTTG-3’  Reverse: 5’-AGGTCTCTTGGTATGTGTTTGCC-3’ |  |
| AurB | Forward: 5’-GTAATAGGCGCGCCATGGCCCAGAAGGAGAA-3’  Reverse: 5’-CATTAAGAATTCTCAGGCGACAGATTGAA-3’ |  |
| BRCA1 | Forward: 5'-AGATGTGTGAGGCACCTGTGG-3’  Reverse: 5'-CACTCTAAGCTCCTGGCACTGGTAGAGG-3’ |  |
| BRCA2 | Forward: 5'-AATGCCCCATCGATTGGTC-3'  Reverse: 5'-AGCCCCTAAACCCCACTTCAT-3', |  |
| GAPDH | Forward: 5’- GGCCTCCAAGGAGTAAGACC-3’  Reverse: 5’-CAAGGGGTCTACATGGCAAC-3’ |  |

**References：**

1. Lee S, Cimica V, Ramachandra N, Zagzag D, Kalpana GV: **Aurora A is a repressed effector target of the chromatin remodeling protein INI1/hSNF5 required for rhabdoid tumor cell survival.** *Cancer Res* 2011, **71:**3225-3235.

2. Gully CP, Velazquez-Torres G, Shin JH, Fuentes-Mattei E, Wang E, Carlock C, Chen J, Rothenberg D, Adams HP, Choi HH, et al: **Aurora B kinase phosphorylates and instigates degradation of p53.** *Proc Natl Acad Sci U S A* 2012, **109:**E1513-1522.

3. Thompson C, MacDonald G, Mueller CR: **Decreased expression of BRCA1 in SK-BR-3 cells is the result of aberrant activation of the GABP Beta promoter by an NRF-1-containing complex.** *Mol Cancer* 2011, **10:**62.

4. Sapir E, Gozaly-Chianea Y, Al-Wahiby S, Ravindran S, Yasaei H, Slijepcevic P: **Effects of BRCA2 deficiency on telomere recombination in non-ALT and ALT cells.** *Genome Integr* 2011, **2:**9.

5. Wang Z, Hou J, Lu L, Qi Z, Sun J, Gao W, Meng J, Wang Y, Sun H, Gu H, et al: **Small ribosomal protein subunit S7 suppresses ovarian tumorigenesis through regulation of the PI3K/AKT and MAPK pathways.** *PLoS One* 2013, **8:**e79117.
