# Supplementary figures and images for: The negative interplay between Aurora A/B and BRCA1/2 controls cancer cell growth and tumorigenesis via distinct regulation of cell cycle progression, cytokinesis, and tetraploidy
Source: Mol Cancer. 2014 Apr 28;13:94. doi: 10.1186/1476-4598-13-94 (PMC4028103; doi:10.1186/1476-4598-13-94)

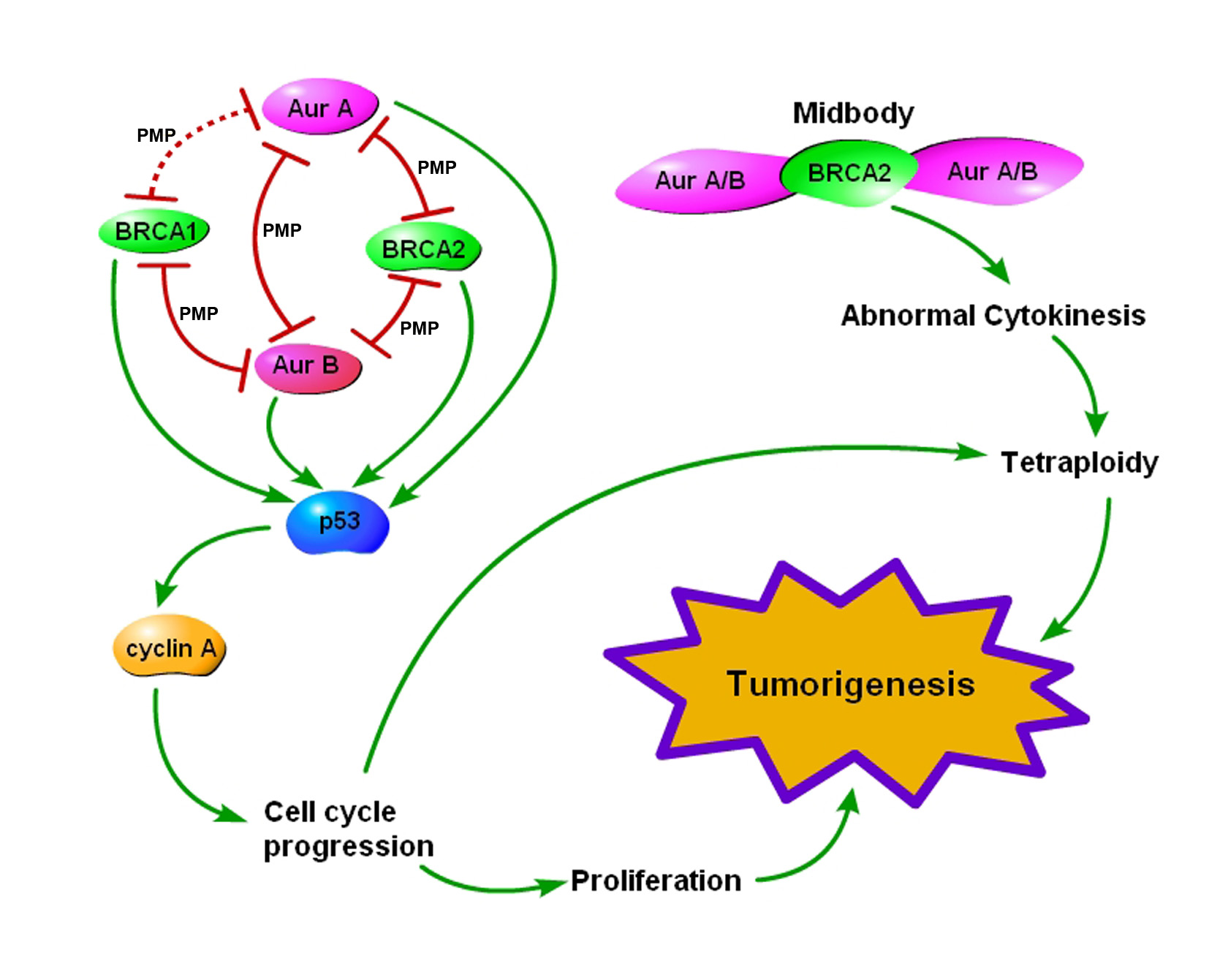

Supplement: Additional file 2: Figure S1 — A schematic diagram illustrates how Aur A/B and BRCA1/2 regulate cell cycle progression and cytokinesis to modulate tetraploidy and tumorigenesis. PMP: proteasome-mediated proteolysis. [file 1476-4598-13-94-S2.jpeg]
